# Smad7 palmitoylation by the S-acyltransferase zDHHC17 enhances its inhibitory effect on TGF-β/Smad signaling

**DOI:** 10.1016/j.jbc.2024.107462

**Published:** 2024-06-12

**Authors:** Oleksandr Voytyuk, Yae Ohata, Aristidis Moustakas, Peter ten Dijke, Carl-Henrik Heldin

**Affiliations:** 1 Department of Medical Biochemistry and Microbiology, Science for Life Laboratory, Box 582, Biomedical Center, Uppsala University, Uppsala, Sweden; 2 Department of Cell and Chemical Biology, Oncode Institute, Leiden University Medical Center, Leiden, The Netherlands

**Keywords:** post-translational modification, palmitoylation, signaling, Smad7, TGF-β

## Abstract

Intracellular signaling by the pleiotropic cytokine transforming growth factor-β (TGF-β) is inhibited by Smad7 in a feedback control mechanism. The activity of Smad7 is tightly regulated by multiple post-translational modifications. Using resin-assisted capture and metabolic labeling methods, we show here that Smad7 is S-palmitoylated in mammary epithelial cell models that are widely studied because of their strong responses to TGF-β and their biological relevance to mammary development and tumor progression. S-palmitoylation of Smad7 is mediated by zDHHC17, a member of a family of 23 S-acyltransferase enzymes. Moreover, we identified four cysteine residues (Cys202, Cys225, Cys415, and Cys417) in Smad7 as palmitoylation acceptor sites. S-palmitoylation of Smad7 on Cys415 and Cys417 promoted the translocation of Smad7 from the nucleus to the cytoplasm, enhanced the stability of the Smad7 protein, and enforced its inhibitory effect on TGF-β-induced Smad transcriptional response. Thus, our findings reveal a new post-translational modification of Smad7, and highlight an important role of S-palmitoylation to enhance inhibition of TGF-β/Smad signaling by Smad7.

Transforming growth factor-β (TGF-β) belongs to a large cytokine family that also includes bone morphogenetic proteins (BMPs), growth and differentiation factors, activins, inhibins and anti-Mullerian hormone; members of this family regulate a variety of cellular processes, including proliferation, apoptosis, and differentiation of many cell types, by binding to two different types of serine/threonine/tyrosine kinase receptors (type I and II receptors) and formation of hetero-tetrameric receptor complexes. After TGF-β binding to its receptors, the constitutively active type II receptor kinase phosphorylates and activates the type I receptor, which in turn phosphorylates receptor-regulated Smads (R-Smads; *i.e.* Smad2 and Smad3) at C-terminal serine residues. Phosphorylated R-Smads then associate with the Co-Smad, Smad4, to form activated Smad complexes, which translocate into the nucleus to regulate the transcription of various specific target genes in cooperation with other transcription factors, as well as co-activators/co-repressors (1, 2). In addition, TGF-β activates a number of non-Smad signaling pathways, including MAP-kinases, phosphatidylinositol 3′-kinase, and Src. TGF-β signaling pathways are tightly regulated, and misregulation of TGF-β signaling promotes the development of various diseases, including fibrosis, autoimmune diseases, and multiple types of cancer (3, 4). Inhibitory Smads (I-Smads; *i.e.* Smad6 and Smad7) are involved in negative feedback control (5, 6, 7). While BMP signaling is inhibited by both Smad6 and Smad7, TGF-β signaling is inhibited preferentially by Smad7 (8, 9). Smad7 inhibits the binding of R-Smads to the receptor, thereby inhibiting type I receptor-mediated R-Smad phosphorylation (10). Moreover, Smad7 recruits HECT-type E3 ubiquitin ligases (11, 12) and the GADD34 phosphatase (13) antagonizing TGF-β signaling by mediating receptor degradation *via* proteasomes and receptor dephosphorylation, respectively. In addition to the inhibitory role of Smad7 in the cytosol, Smad7 has been reported to directly bind to DNA and inhibit the transcription of genes in the nucleus (14). The activity of Smad7 has been shown to be tightly regulated in cells through a number of mechanisms, including post-translational modifications (PTMs), such as methylation (15, 16), ubiquitination (12, 17, 18), acetylation (19) and phosphorylation (20).

Among the PTMs, many proteins are modified by the attachment of lipid moieties, such as myristoylation, prenylation, and palmitoylation (also known as S-acylation), which can modulate protein localization, activity, and stability (21). S-acylation is unique among lipid modifications since it is the only known reversible form of lipid modification (22). It involves the addition of acyl chains to cysteine residues through thioester bonds, altering the hydrophobicity of the proteins thereby controlling their sorting, stability, interaction with membranes, and activity (23, 24). Additionally, S-acylation is crucial for signaling *via* several cell surface receptors (25, 26, 27, 28). This PTM is frequently referred to as S-palmitoylation, reflecting the fact that palmitate is the predominant fatty acid attached to proteins through thioester bonds (29).

In mammalian cells, S-palmitoylation is dynamically regulated by protein acyl-transferases (PATs) and thioesterases (23, 30). The 23 human PATs belonging to the “zDHHC” family share similar structures, encompass four to six transmembrane domains and a highly conserved 51-amino acid zinc finger Asp-His-His-Cys (zDHHC) motif, embedded within a cysteine-rich domain (CRD). The catalytic domain of these enzymes (zDHHC-CRD) is positioned at the cytosol-membrane interface, which allows them to perform S-palmitoylation in close proximity to the cell membrane or other vesicular organelles (31).

Dad, the single *Drosophila* homolog of I-Smads, has been shown to be S-palmitoylated (32). In the present study, we show that also mammalian Smad7 is palmitoylated, exhibiting a more complex pattern of cysteines that serve as sites for this PTM. We have also investigated the functional consequences of this modification.

## Results

### Smad7 is S-acylated by endogenous protein S-acyltransferases (PATs)

**Figure 1:**
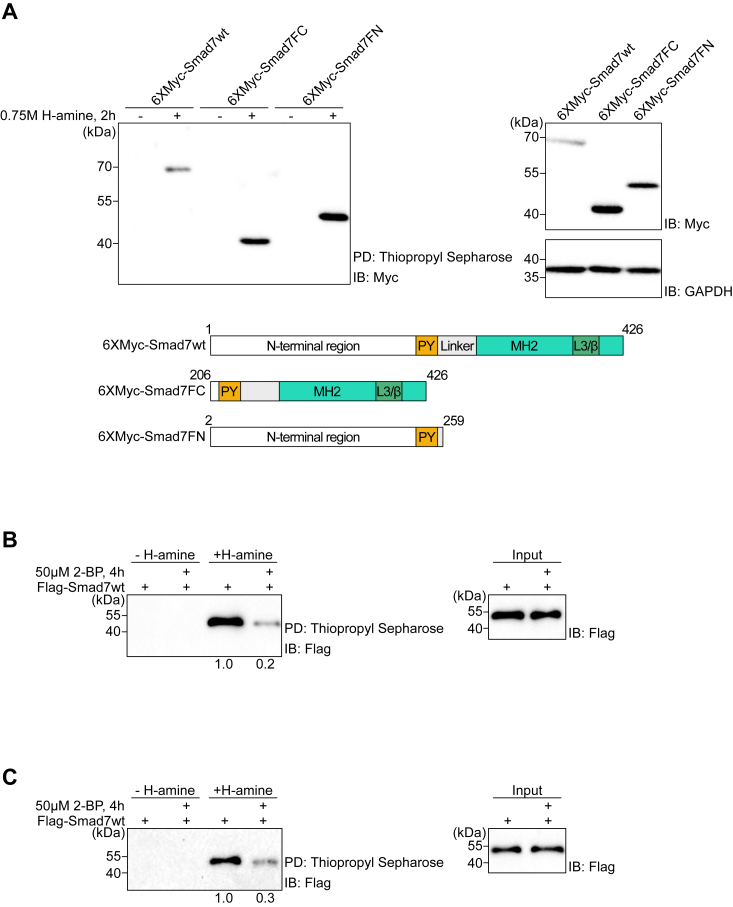
**Smad7 is S-acylated by endogenous PATs.***A*, HEK293T cells were transfected with plasmids encoding wt or deletion mutants of Myc-Smad7 (*lower panel*). Cells were lysed and lysates analyzed by the acyl-RAC assay, followed by immunoblotting (IB) with a Myc antibody. Lysis Buffer was used as a negative control, in which thioesters are not cleaved. The *right* panel shows the expression levels of wt and mutant Smad7. GAPDH was included as loading control. *B* and *C*, MCF10A cells (*B*) and MDA-MB-231 cells (*C*) were transfected with Flag-Smad7 and treated with or without 50 μM of the palmitoylation inhibitor 2-BP. Cell lysates were subjected to immunoprecipitation (IP) with a Flag antibody; immobilized proteins were eluted from the beads, diluted in lysis buffer (input), and subjected to acyl-RAC assay, followed by IB with a Flag antibody. For quantification, palmitoylation signals were normalized to the corresponding input signals; the control sample (not treated with 2-BP), was set at 1.0. Each experiment was repeated three times and the result of representative experiments are shown. L3/β, loop 3, β-strand; MH2, Mad homology 2; PY, proline-rich tyrosine motif.

Since the *Drosophila* homolog of inhibitory Smad, Dad, was reported to be palmitoylated (32), we investigated whether Smad7 is S-acylated also in mammalian cells. The well-established instability and low expression of Smad7 in mammalian cells (10, 11, 19), led us to rely on expression of transfected Smad7 in these assays. To detect S-acylated Smad7, we performed an acyl resin-assisted capture (Acyl-Rac) assay in human embryonic kidney HEK293T cells (Fig. 1*A*). The Acyl-Rac assay removes acyl groups from cysteine residues with hydroxylamine, followed by direct conjugation of the free cysteines by a thiol-reactive resin (33). In the presence of hydroxylamine, wild-type (wt) Smad7, as well as truncation mutants containing the C- and N-terminal parts of Smad7, respectively, were detected in the thiol-reactive resin pull-down fraction, but not in the negative control that was not treated with hydroxylamine (Fig. 1*A*). This result suggests that S-acylation of Smad7 occurs on cysteine residues in both N- and C-terminal parts of Smad7, by endogenous enzymes. To determine whether Smad7 is palmitoylated also in normal and malignant breast epithelial cells, we analyzed the human breast epithelial cell line MCF10A (Fig. 1*B*) and the human triple-negative breast cancer cell line MDA-MB-231 (Fig. 1*C*). These two cell models are widely studied due to their potent response to TGF-β signaling and their potential to form mammary organoids (MCF10A) and contribution to breast cancer progression and metastasis (MDA-MB-231). In order to rule out the possibility of an indirect S-acylation of Smad7, we immunoprecipitated Smad7 in both cell lines and eluted proteins from the beads in a buffer containing 1% SDS to dissociate protein-protein interactions (Fig. 1, *B* and *C*); the eluent was then diluted and subjected to the Acyl-Rac assay. We detected S-acylated Smad7 in both cell lines in the presence of hydroxylamine (H-amine) but not in the control. Treatment with the general palmitoylation inhibitor 2-bromopalmitate (2-BP), which is known to both inhibit palmitoylation and de-palmitoylation, effectively inhibited Smad7 S-acylation (Fig. 1, *B* and *C*). Taken together, these findings support the notion that Smad7 is S-acylated in mammalian cells.

### Smad7 interacts with zDHHC17

In humans, S-acylation is performed by 23 members of the zDHHC family of enzymes. To identify potential interactions between PATs and Smad7, we first performed a co-immunoprecipitation assay, using HEK293 cells which have a high transfection efficiency. Since a given mammalian cell does not express all 23 PATs, we prioritized a forward, gain-of-function approach using vector expression of all 23 human PATs. Co-expression of each of the 23 HA-tagged zDHHC PATs (34) with Flag-Smad7, followed by pull-down with anti-HA magnetic beads, and immunoblotting with a Flag antibody, revealed co-immunoprecipitation of Smad7 with zDHHC3, 5, 7, 9, 12, 13, 14, and 17 (Fig. S1). zDHHC3 and zDHHC7 are considered to be low-selectivity/high-activity isoforms, which are active against a plethora of proteins and do not appear to recognize specific features of their substrates to mediate S-acylation, whereas zDHHC13 and zDHHC17 are high-selectivity/low-activity enzymes which require specific recognition of their substrate proteins for successful S-acylation (35). These results support the notion that Smad7 may be a substrate for several PATs, but since zDHHC17 is the human homolog of palmitoyl acyl-transferase dHIP14 which catalyzes Dad palmitoylation in *Drosophila* (32), we decided to explore further the role of zDHHC17 in Smad7 S-acylation.

**Figure 2:**
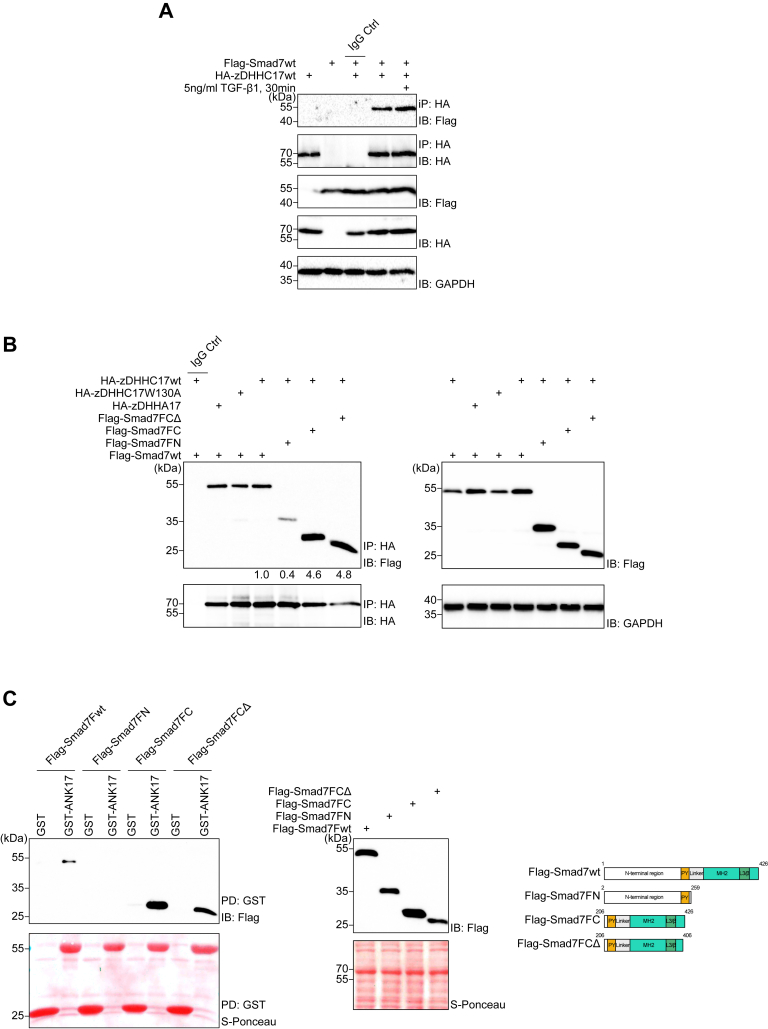
**Smad7 interacts with zDHHC17.***A*, HEK293T cells transfected with indicated plasmids were treated with 5 ng/ml TGF-β1 for 30 min or *left* untreated. Cell lysates were subjected to IP using an HA antibody or control IgG, followed by IB with Flag or HA antibodies. The *top two panels* show the interaction and immunoprecipitation efficiency, and the *lower three panels* show the expression of each protein. GAPDH was included as a loading control. *B*, lysates from HEK293T cells transfected with the indicated constructs of Smad7 and zDHHC17 were subjected to IP with an HA antibody, followed by IB with Flag or HA antibodies. The *left panel* shows the interaction and immunoprecipitation efficiency and the *right panel* shows the expression of each protein. GAPDH was included as loading control. For quantification, Flag signals were normalized to the corresponding HA signals in the IP panel; co-immunoprecipitation with overexpressed Flag-Smad7wt and zDHHC17wt, was set at 1.0. *C*, lysates from HEK293T cells, transfected with the indicated constructs of Smad7, were incubated with either GST-ANK17 or GST and glutathione beads. Total cell lysates (TCL) and proteins pulled down were subjected to SDS–PAGE and transferred to nitrocellulose membranes, followed by Ponceau-S staining, and IB analysis using the antibodies indicated. The *right panel* illustrates the structures of the Smad7 deletion mutants which were used for co-immunoprecipitation and GST pulldown assays. Each experiment was repeated three times and the results of representative experiments are shown.

To confirm an interaction between Smad7 and zDHHC17, we used hemagglutinin (HA)-tagged zDHHC17 to capture Flag-tagged wt Smad7 in HEK293T cell lysates and found that zDHHC17 was co-immunoprecipitated with Smad7; the binding of Smad7 to zDHHC17 was slightly increased upon stimulation with TGF-β for 30 min (Fig. 2*A*). To identify the region of Smad7 that is involved in interaction with zDHHC17, we examined Smad7 truncation mutants lacking the N- or C-terminals. We also used a mutant containing the C-terminal part, but lacking the last 20 amino acid residues (Fig. 2*C*, right panel), since this segment is conserved between the *Drosophila* Dad protein and the human Smad7, and since it was found to be palmitoylated in Dad (32). By co-immunoprecipitation, we found that zDHHC17 preferentially bound to the C-terminal part of Smad7, and less to the N-terminal part (Fig. 2*B*). zDHHC17 bound much stronger to the C-terminal truncation mutant of Smad7 than to wt Smad7, which could be explained by the known fact that the N-terminal part of Smad7 physically interacts with the MH2 domain in the C-terminal part of Smad7 (8) and thus may mask the epitope that mediate the interaction with zDHHC17. Verardi *et al.* (36) previously reported that Trp130 (W130) is critical for the interaction of the ankyrin repeat domain (ANK17) in zDHHC17 with the ANK binding motif (zDABM) of other proteins. We therefore analyzed whether a W130A mutant of zDHHC17, as well as a catalytically inactive C467A zDHHC17 mutant, designated HA-zDHHA17, bound to Smad7. We found that both mutants bound Smad7 to the same extent as wt zDHHC17 (Fig. 2*B*).

It has been previously shown that the essential component for neuronal exocytosis pathways, synaptosome-associated protein 25 (SNAP25b) and a vesicular protein located in the synapses, cysteine string protein α (CSPα), although being S-acylated by many Golgi zDHHC enzymes (37), are specifically recruited by the ankyrin repeat domain of zDHHC17 (35). Despite the facts that Smad7 does not contain a classical zDABM motif and that the canonical substrate-binding pocket in ANK17, containing W130, is not required for Smad7 binding (Fig. 2*B*), we investigated whether ANK17 is sufficient for binding to Smad7. Indeed, in a GST pull-down assay, we were able to show an interaction between ANK17 and wt Smad7; this interaction preferentially involved the C-terminal part of Smad7 (Fig. 2*C*). These observations suggest that the ANK17 domain of zDHHC17 binds the C-terminal part of Smad7 and that the catalytical activity of zDHHC17 is not required for binding.

### zDHHC17 promotes Smad7 palmitoylation

**Figure 3:**
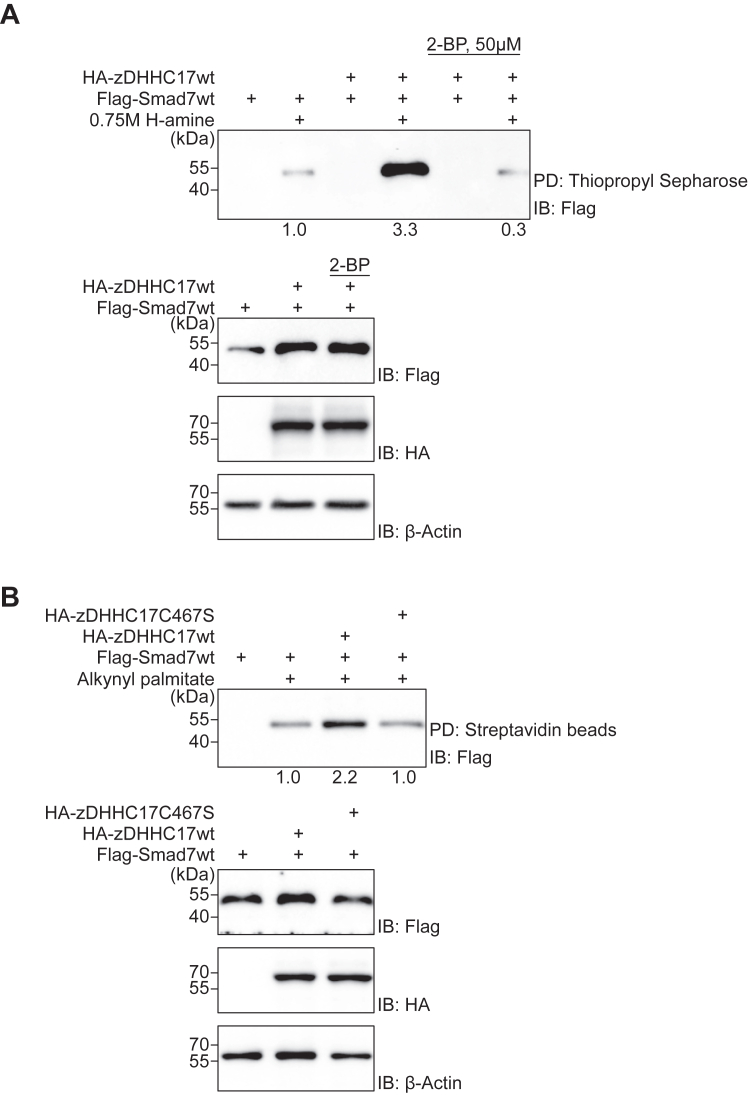
**zDHHC17 increases Smad7 palmitoylation.***A*, HEK293T cells were transfected with plasmids encoding the indicated proteins and treated with or without 50 μM 2-BP. Cell lysates were analyzed by the acyl-RAC assay, followed by IB using a Flag antibody. *Lower panels* show protein expression levels. β-Actin was included as a loading control. For quantification, palmitoylation signals in the *upper panel* were normalized to the corresponding Flag signals in the total cell lysate (*lower panel*); signal with overexpressed Flag-Smad7wt alone, was set at 1.0. *B*, HEK293T cells were transfected with Flag-tagged wt Smad7, together with either pEF-BOS-HA-tagged wt zDHHC17 or HA-tagged zDHHC17C467S (catalytically inactive enzyme). Click reaction was performed and samples were subjected to pull-down with Streptavidin magnetic beads followed by IB using a Flag antibody. *Lower panels* show the expression of each protein, as indicated. β-Actin was included as a loading control. For quantification, palmitoylation signals in the upper panel were normalized to the corresponding Flag signals in the total cell lysate (*lower panel*); signal with overexpressed Flag-Smad7wt alone, was set at 1.0. Each experiment was repeated three times and the results of representative experiments are shown.

We further investigated whether Smad7 is a substrate for zDHHC17 by performing the Acyl-Rac assay using transfected HEK293T cells. In cells expressing wt zDHHC17, significantly more Smad7 was detected in the thiol-reactive resin pull-down fraction, compared to cells transfected with empty vector; treatment of cells overexpressing wt zDHHC17 with 2-BP effectively inhibited S-acylation of Smad7 by wt zDHHC17 (Fig. 3*A*). These findings suggest that Smad7 is S-acylated through an enzymatic process performed by the S-acyltransferase zDHHC17.

S-acylation is commonly referred to as palmitoylation, reflecting the fact that palmitic acid is the predominant fatty acid added to proteins (29). To determine whether the S-acylation of Smad7 was specifically S-palmitoylated, we metabolically labeled cells with alkynyl palmitic acid; using a click chemistry reaction, we found that overexpression of wt zDHHC17 dramatically increased the S-palmitoylation of Smad7, whereas a catalytically inactive mutant of zDHHC17 (HA-zDHHC17C467S) was not able to palmitoylate Smad7 to the same level as wt zDHHC17 (Fig. 3*B*). These observations suggest that the catalytic activity of zDHHC17 mediates an appreciable Smad7 palmitoylation.

By using the same approach with metabolic labeling of cells, followed by a click chemistry reaction, we were unable to detect any palmitoylation of Smad2, Smad3, Smad4, and Smad6, or the type I (also known as activin receptor-like kinase 5, ALK5) and type II TGF-β receptors by zDHHC17 (Fig. S2). These findings suggest that the S-acyltransferase zDHHC17 selectively palmitoylates Smad7 in the TGFβ signaling pathway.

### zDHHC17 palmitoylates four cysteine residues in Smad7

In order to determine in which Smad7 domain palmitoylation occurs, Smad7 deletion mutants (Fig. S3*A*) were ectopically expressed together with wt zDHHC17. Click chemistry reaction assay revealed that zDHHC17 S-palmitoylated Smad7 preferentially in the C-terminal part, which includes the so-called linker and the Mad homology 2 (MH2) domains (Fig. S3, *A* and *B*). Mutation of all individual C-terminal cysteine residues revealed that S-palmitoylation was decreased after mutation of Cys225, Cys415, and Cys417 in the C-terminal part of Smad7 (Fig. S3*B*).

**Figure 4:**
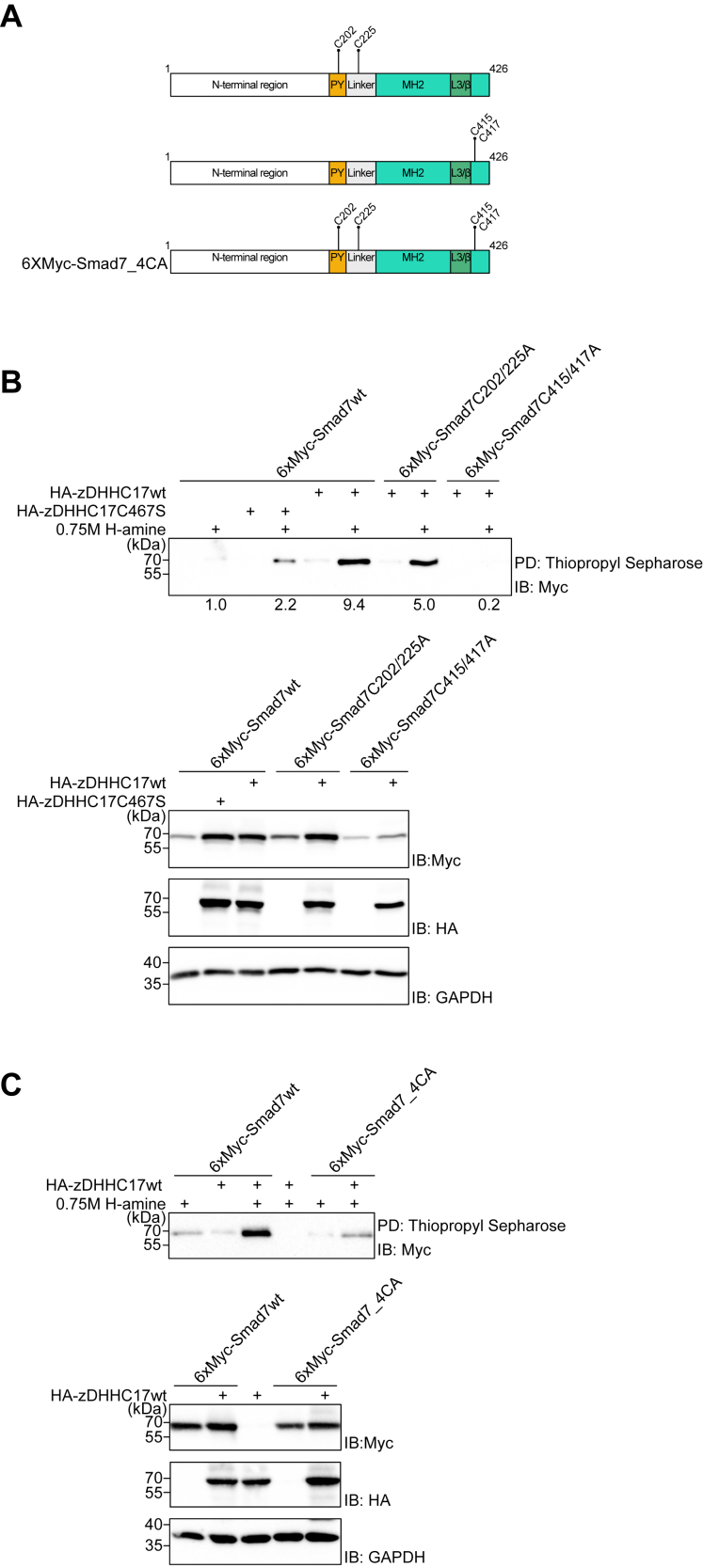
.**zDHHC17 palmitoylates four cysteine residues in Smad7.***A*, schematic representation of Smad7 cysteine mutants used in the Acyl-RAC assay. *B* and *C*, HEK293T cells were transfected with plasmids encoding wt Smad7 or the indicated Smad7 cysteine mutants, together with HA-tagged wt zDHHC17 or zDHHC17C467S mutant (catalytically inactive enzyme; *B*). Cell lysates were analyzed by the acyl-RAC assay followed by IB using a Myc antibody. Lower panels show protein expression levels. GAPDH was included as a loading control. For quantification, palmitoylation signals in the *upper panel* (*B*) were normalized to the corresponding Myc signals in the total cell lysate (*lower panel*s); signal with overexpressed Myc-Smad7wt alone, was set at 1.0. The experiment was repeated three times and the result of a representative experiment is shown.

In order to exclude potential artifacts caused by the lower expression of the N-terminal domain (Flag-Smad7FN; Fig. S3*B*), we also mutated all cysteines located in the N-terminal part of Smad7 in the context of the full-length protein and found that only the Cys202 mutant showed less S-palmitoylation in the presence of zDHHC17 (Fig. S3*C*). In order to further analyze the palmitoylation of Smad7, we studied Smad7 mutants in which either the two linker region cysteine residues, C202 and C225, or the two C-terminal MH2 cysteine residues, C415 and C417, were mutated in the context of full-length Smad7 (Fig. 4*A*). These mutants were then co-expressed with wt zDHHC17; in addition, Smad7 was also co-expressed with catalytically inactive zDHHC17C467S, as an additional negative control (Fig. 4*B*). The C202/225A Smad7 mutant showed a significantly decreased S-palmitoylation by zDHHC17wt, although the S-palmitoylation level was higher than the level seen after transfection of the catalytically inactive zDHHA17 mutant together with wt Smad7; on the other hand, the C415/417A mutant showed a dramatic decrease in the S-palmitoylation level (Fig. 4*B*; note that this double mutant was expressed at lower levels than the other Smad7 proteins). As expected, mutation of all four cysteine residues implicated in palmitoylation, *i.e.* C202/225/415/417A resulted in an even more pronounced decrease in S-palmitoylation after overexpression of wt zDHHC17 (Fig. 4*C*). Taken together, two linker region cysteine residues (Cys202 and Cys225) and two C-terminal cysteine residues (Cys415 and Cys417) in the MH2 domain are key residues required for efficient S-palmitoylation of Smad7 by zDHHC17.

### Palmitoylation affects Smad7 subcellular localization

**Figure 5:**
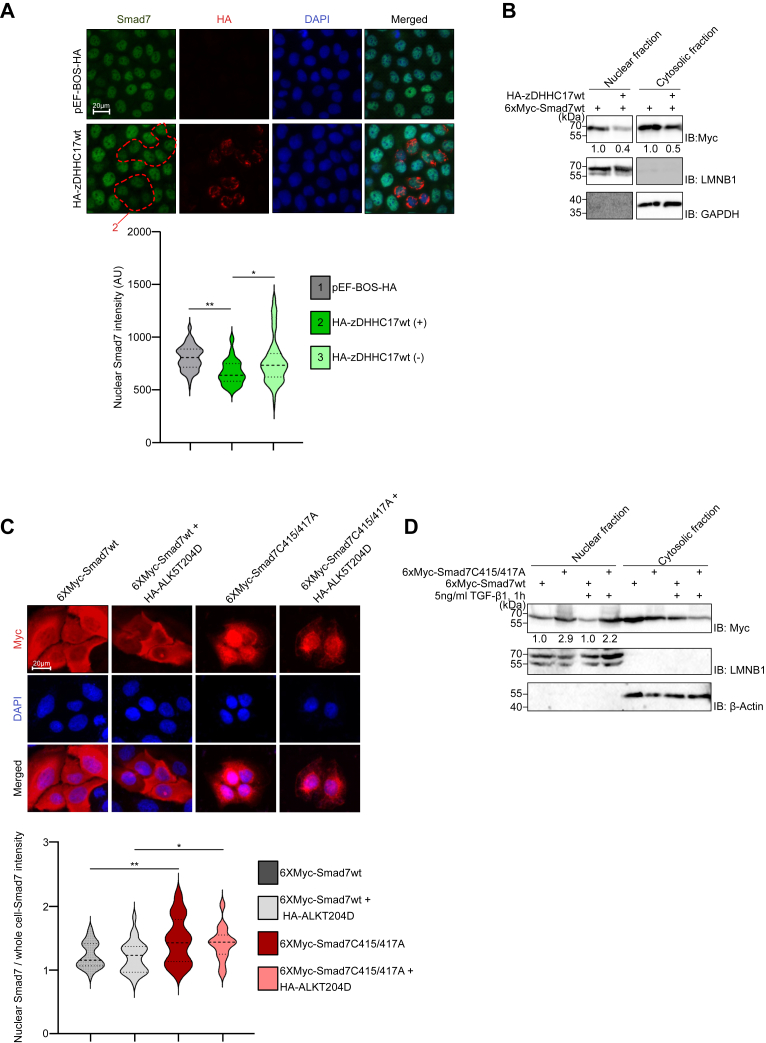
**Palmitoylation affects Smad7 subcellular localization.***A*, subcellular localization of Smad7. HaCaT cells were transfected with either empty vector pEF-BOS-HA or HA-tagged wt zDHHC17. Cells were then stained with antibodies against Smad7 (*green*), HA-zDHHC17 (*red*), and with DAPI for nuclear staining (*blue*). The nuclear score of Smad7 in 110 to 128 cells transfected or not with HA-zDHHC17 is presented graphically using color-coded violin plots along with confidence intervals and statistical significance (*lower panel*). The data represent three independent biological experiments. Data shown are mean ± SD of at least 20 fields of view (n ≥ 20) from one representative biological experiment. Each biological experiment included three technical repeats. Statistical significance was assessed using one-way ANOVA followed by Tukey’s multiple comparisons or two-tailed student *t* test. ∗*p* < 0.05; ∗∗*p* < 0.01. *B*, HaCaT cells were transfected with the indicated combinations of plasmids. At 24 h post transfection, cell lysates were fractionated and IB was performed to analyze the presence of Myc-tagged wt Smad7, laminB1 (LMNB1, nuclear marker) and GAPDH (cytoplasmic marker). For quantification, we normalized Myc signals in the nucleus and cytoplasmic fractions to LaminB1 and GAPDH, respectively. Signals from samples with overexpressed Myc-Smad7wt alone were set at 1.0. The experiment was repeated three times and the result of a representative experiment is shown. *C*, HaCaT cells were transfected with either 6xMyc-wt Smad7 or a 6xMyc Smad7 C415/417A mutant together with HA-ALK5 T204D (constitutively active receptor) or control vector. Cells were stained with anti-Myc antibodies for Smad7 (*red*); nuclear staining by DAPI (*blue*) is also shown. The nuclear score of Smad7 normalized to total score of Smad7 in 29 to 32 cells is presented graphically using color-coded violin plots along with confidence intervals and statistical significance (*lower panel*). The data represent three independent biological experiments. Data shown are mean ± S.D. of at least 20 fields of view (n ≥ 20) from one representative biological experiment. Each biological experiment included three technical repeats. Statistical significance was assessed using one-way ANOVA followed by Tukey's multiple comparison or two-tailed student *t* test. ∗*p* < 0.05; ∗∗*p* < 0.01. *D*, HaCaT cells were transfected with the indicated combinations of plasmids. At 24 h post transfection, cells were starved for 16 h and treated with 5 ng/ml TGF-β1 for 1 h or *left* untreated. Cell lysates were fractionated and IB was performed to analyze the presence of Myc-tagged Smad7. For quantification, we normalized the Myc signals in the nuclear fraction to LaminB1; signal from samples with overexpressed Myc-Smad7wt was set at 1.0. The experiment was repeated three times and the result of a representative experiment is shown.

Since palmitoylation has been shown to affect the subcellular localization and trafficking of other proteins (33, 38), we investigated whether S-palmitoylation altered the subcellular distribution of Smad7, using human keratinocyte (HaCaT) cells which respond well to TGF-β and are suitable for this type of experiments. In the absence of transfected wt zDHHC17, Smad7 was more located in the nucleus compared to Smad7 S-palmitoylated by zDHHC17, as determined by immunofluorescence staining experiments. The cytoplasmic localization of Smad7 correlated with the cytoplasmic localization of zDHHC17, which formed specific punctuated patterns some of which overlapped with the Golgi apparatus, and other clusters were dispersed in the cytoplasm (Fig. 5*A*). To illustrate this, the fluorescence intensity of endogenous Smad7 in the nucleus was calculated (Fig. 5*A*). The differential localization was statistically significant as analyzed by a quantitative calculation followed by ANOVA test and Tukey's multiple comparisons test. To confirm this finding, we performed subcellular fractionation of HaCaT cells, followed by immunoblot analysis. As shown in Figure 5*B*, in cells overexpressing zDHHC17 the nuclear fraction contained less Smad7 than in cells not transfected with zDHHC17. Moreover, the C415/417A Smad7 mutant, which showed decreased S-palmitoylation, was located more in the nucleus compared with wt Smad7, with or without TGF-β stimulation, as determined by both immunofluorescence staining (Fig. 5*C*) and subcellular fractionation (Fig. 5*D*) experiments. Note that two alternative methods of TGF-β stimulation were analyzed, *that is*, co-transfection of a constitutively active type I receptor (ALK5T204D; Fig. 5*C*), and stimulation of the cells with 5 ng/ml TGF-β (Fig. 5*D*), providing the same result.

Taken together, these results show that S-palmitoylation of Cys415 and Cys417 leads to less nuclear localization of Smad7.

### C-terminal S-palmitoylation of Smad7 affects its stability

**Figure 6:**
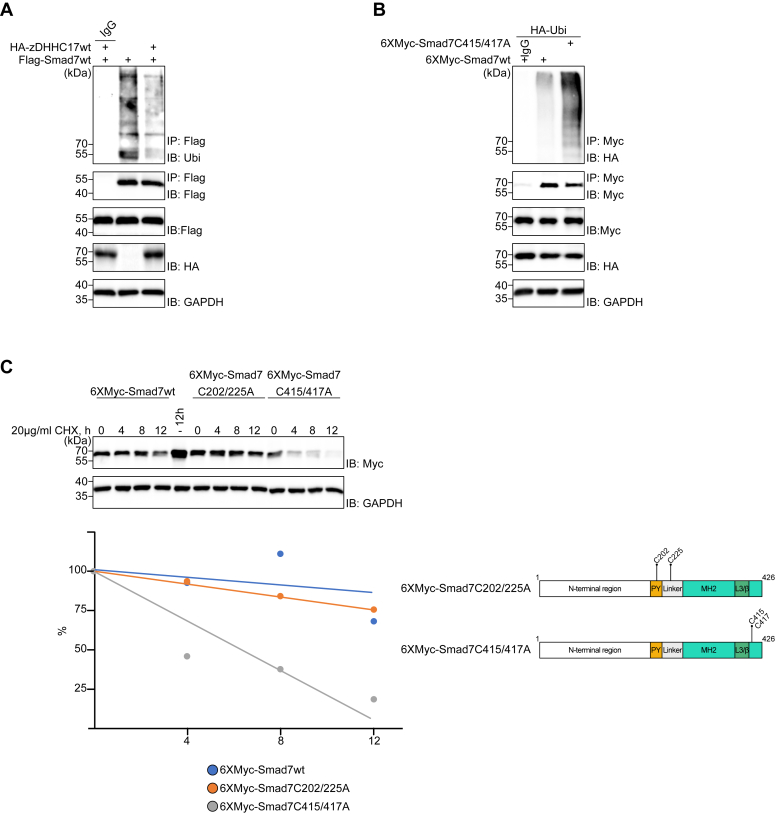
**C-terminal palmitoylation affects Smad7 ubiquitination and stability.***A* and *B*, HEK293T cells were transfected with the indicated plasmids, and treated with 10 μM of MG132 for 4 h before cell lysis. Lysates from cells were subjected to IP using a FLAG antibody (or IgG control) followed by IB using antibodies against ubiquitin or Flag (*A*) or subjected to IP using a Myc antibody (or IgG control) followed by IB using antibodies against HA or Myc (*B*). *Lower panels* show the expression of each protein, as indicated. GAPGH was included as a loading control. *C*, HEK293T cells were transfected with the indicated plasmids, and treated with 20 μg/ml of cycloheximide for the indicated time periods. Cells were lysed in an SDS-sample buffer and subjected to IB using antibodies against Myc and GAPDH (loading control). Quantification of Myc/GAPDH signal was performed and used to estimate the half-life of each protein (*lower left part of the panel*). Structures of Smad7 with point mutations are shown (*lower right panel*). Each experiment was repeated three times and the results of representative experiments are shown.

Poly-ubiquitination of TGF-β receptors (39, 40) and Smad molecules (17, 41) have been reported to induce proteasomal degradation of these proteins. Therefore, we performed a ubiquitination assay, to determine whether S-palmitoylation of Smad7 affects its ubiquitination in HEK293T cells. As shown in Figure 6*A*, the brisk polyubiquitination of Smad7 was suppressed by overexpression of zDHHC17. In contrast, the C415/417A Smad7 mutant with decreased palmitoylation, was poly-ubiquitinated significantly stronger than wt Smad7 (Fig. 6*B*). We then analyzed whether the Smad7 ubiquitination mediated its degradation. Analysis by immunoblotting of cells treated with cycloheximide in order to inhibit protein synthesis for various time periods, revealed that the half-life of the C415/417A mutant Smad7 was significantly shorter compared to that of wt Smad7 or the C202/225A Smad7 mutant (Fig. 6*C*).

Our results confirmed the previous findings reporting that Smad7 is ubiquitinated and support the notion that S-palmitoylation suppresses ubiquitination and degradation through the ubiquitin-proteasome pathway.

### zDHHC17-mediated S-palmitoylation of Smad7 affects transcriptional activities of TGF-β signaling

**Figure 7:**
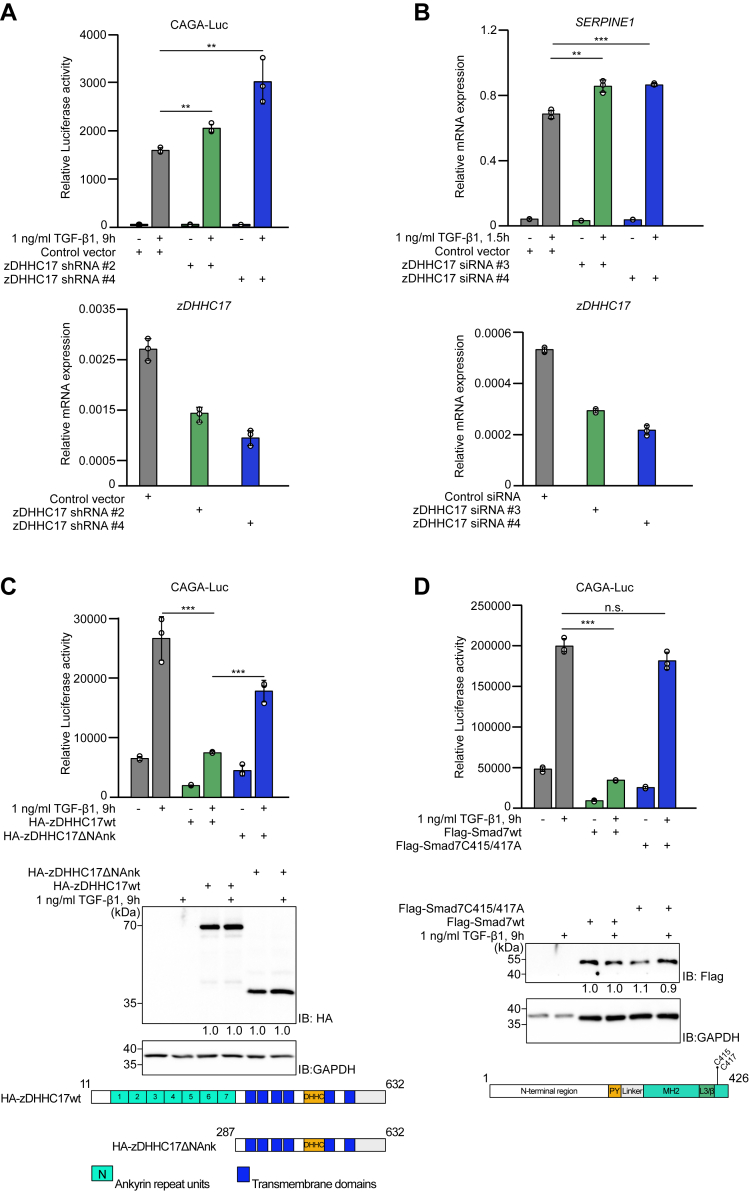
**zDHHC17 affects transcriptional activities of TGF-β signaling.***A*, lentiviral particles containing the indicated shRNAs were used to infect MDA-MB-231 cells to knock down zDHHC17; the level of zDHHC17 after knockdown is shown in the *lower panel*. The expression of *zDHHC17* mRNA was normalized to the expression of the housekeeping gene *GAPDH*. After infection and cell growth in selective media, MDA-MB231 were transfected with the 9xCAGA Luc plasmid, serum-starved for 16 h, stimulated for 9 h with 1 ng/ml TGFβ1, or not, and analyzed by the luciferase assay. *B*, MCF10A cells were transfected with non-targeting siRNA and two *zDHHC17* siRNAs; the level of *zDHHC17* mRNA after knock down is shown in the *lower panel*. Forty-8 hours after transfection, MCF10A cells were serum-starved for 16 h, stimulated with TGF-β1 (1 ng/ml) for 1.5 h, or not, and *SERPINE1* mRNA expression analyzed by qRT-PCR analysis. The expression of *SERPINE1* was normalized to the mRNA expression of *GAPDH*. *C* and *D*, COS7 cells were co-transfected with the 9xCAGA Luc plasmid and indicated cDNAs. Six hours after transfection, cells were serum-starved for 16 h, stimulated for 9 h with 1 ng/ml TGFβ1, or not, and analyzed by the luciferase assay. To quantify protein expression, we normalized HA (*C*) and Flag (*D*) to GAPDH in the total cell lysates. Expression of HA-zDHHC17wt (*C*) and Flag-Smad7wt (*D*) were set at 1.0. Schematic representation of constructs are demonstrated in the *lower panels*. Each experiment was repeated three times and each contained three technical repeats; the result of representative experiments are shown. Data are mean ± SD and error bars denote standard deviation (n = 3). Statistical significance was assessed using unpaired two-tailed student *t* test. ∗*p* < 0.05; ∗∗*p* < 0.01; ∗∗∗*p* < 0.001; n.s. = not significant.

Smad7 has been established as a pivotal negative regulator of TGF-β signaling in diverse cell types (42, 43). We, therefore, investigated whether Smad7 S-palmitoylation affected the inhibitory role of Smad7 in TGF-β/Smad signaling using three distinct cell models in order to include a diverse cellular profile. We first knocked down the palmitoylation enzyme zDHHC17 in MDA-MB231 cells by using lentiviral infection with several shRNA constructs. Each shRNA gave more than 50% knock-down of zDHHC17 (Fig. 7*A*, lower panel). We then performed transcriptional assays using the Smad-responsive CAGA-luciferase reporter (44), with or without knock-down of zDHHC17, in MDA-MB-231 cells in which the CAGA-luciferase reporter shows robust response to TGF-β signaling. As shown in Figure 7*A* (upper panel), the TGF-β-induced CAGA luciferase activity was significantly increased in cells in which zDHHC17 had been knocked down. Note that although the degree of silencing was around 45 to 50%, the biological impact was highly significant, and the independent shRNAs exhibited effects corresponding to the degree of zDHHC17 silencing (Fig. 7*A*). Smad7 has been demonstrated to inhibit TGF-β-dependent expression of the *SERPINE1* gene (encoding plasminogen activator inhibitor 1, PAI1) (14). To address whether S-palmitoylation has an effect on this process, we knocked down zDHHC17 in MCF10A cells by using two different siRNAs (Fig. 7*B*, lower panel), and examined the expression of *SERPINE1*. The TGF-β-enhanced expression of this gene was significantly elevated in cells in which the palmitoylation enzyme zDHHC17 was knocked down, despite the fact that only partial silencing of zDHHC17 was achieved. Based on these results, we conclude that the S-palmitoylation activity of zDHHC17 inhibits TGF-β-dependent transcription in cells, most likely by enhancing the inhibitory effect of Smad7.

Next, we studied the role of overexpressed zDHHC17 on TGF-β-dependent promoter activity. We overexpressed different constructs of zDHHC17 in COS7 cells (Fig. 7*C*, two lower panels), which like HEK293 cells show high transfection efficiency but are more sensitive than HEK293 cells to TGF-β stimulation, and performed the CAGA-luciferase reporter assay. As shown in Figure 7*C* (upper panel), the TGF-β-induced activation of the CAGA-luciferase reporter was suppressed by overexpression of wt zDHHC17. The basal promoter activity was also suppressed by overexpression of wt zDHHC17, which is likely to be due to an effect on endogenously produced TGF-β, since no difference was seen in the presence of a type I receptor kinase inhibitor (O.V., unpublished data). In contrast, overexpression of the zDHHC17ΔNAnk construct, which led to lower S-palmitoylation level of Smad7 (Fig. S4), showed less inhibitory effect (Fig. 7*C*). To consolidate the above findings, we then examined whether the S-palmitoylation level of Smad7 affected its inhibitory effect on TGF-β-dependent CAGA-luciferase reporter activity (44). As expected, wt Smad7 significantly inhibited the promoter activity after stimulation with TGF-β, whereas the Smad7 C415/417A mutant did not (Fig. 7*D*, upper panel). Together, these observations demonstrate that S-palmitoylation of Smad7 by zDHHC17 promotes its inhibitory effect on TGF-β/Smad dependent transcriptional activity.

## Discussion

I-Smads (Smad6 and Smad7) are of key importance for maintaining a proper physiological response to TGF-β and BMP signaling and their deregulation has been linked to a number of different diseases, including fibrosis and cancer (45, 46, 47, 48). The expression level and activity of Smad7 are under tight control of PTMs, including phosphorylation, ubiquitination, methylation and acetylation (16, 17, 19, 20, 49). In this study, we have explored the role of S-palmitoylation, a reversible posttranslational lipid modification of proteins, in the regulation of the stability and activity of Smad7. We report that human Smad7 is S-palmitoylated on two cysteines in the linker domain (C202 and C225) and two C-terminal cysteines in the MH2 domain (C415 and C417). We show that zDHHC17-induced S-palmitoylation of Smad7 promotes the stability and cytoplasmic localization of Smad7. The Smad7 C415/417A mutant was unable to inhibit TGF-β-dependent transcriptional activities, despite the more nuclear enrichment of this mutant, suggesting that C-terminal S-palmitoylation is critical for the inhibitory function of Smad7.

Unlike other lipid posttranslational modifications, S-palmitoylation is dynamically regulated by protein acyltransferases and thioesterases. In human cells, S-palmitoylation is catalyzed by 23 members of the zDHHC family. It has been demonstrated that PATs are able to palmitoylate a number of substrates, including cytosolic proteins and cell surface receptors (25, 26, 32, 50, 51, 52). We identified zDHHC17 as a PAT that binds to and selectively, among other Smad proteins and TGF-β receptors, palmitoylates Smad7 in the TGF-β signaling pathway. Since Smad7 was found to interact with several additional PATs under conditions of overexpression, it would be interesting to investigate whether Smad7 is palmitoylated also by any of these members of the zDHHC family. Although we identified Smad7 as the only TGF-β receptor and Smad member to be S-palmitoylated by zDHHC17 (Fig. S2), it remains to be elucidated whether other PATs are able to S-palmitoylate other proteins involved in TGF-β signaling. zDHHC17 binds its substrates preferentially through its N-terminal ankyrin repeats (53). We confirmed that this is the case also for Smad7 by showing an interaction between the N-terminal ankyrin repeats of zDHHC17 and the C-terminal linker-MH2 domains of Smad7. Moreover, we demonstrated that the catalytic activity of zDHHC17 is not needed for the interaction between zDHHC17 and Smad7, and neither was the interaction dependent on W130, an essential residue for the binding of zDHHC17 to other substrates (36). This could be explained by the fact that Smad7 does not contain a classical zDHHC ankyrin-repeat binding motif (zDABM). Our data are in line with the findings of Butler *et al.* (52), who reported that an interaction between zDABM and ankyrin repeats of zDHHC17 does not occur for all S-palmitoylated proteins. An important finding from this study is the mode of regulation of TGF-β signaling by zDHHC17. The strictly cytoplasmic localization of zDHHC17 is consistent with the finding that its presence mobilized Smad7 to the cytoplasm. The export of Smad7 from the nucleus by binding to Smurf proteins in response to TGF-β signaling is well established (11, 54). It was shown that Smurf1/2, upon translocation from the nucleus in complex with Smad7, binds to the type I TGF-β receptor, inducing its ubiquitination and degradation, leading to suppression of TGF-β signaling (11, 54). In agreement with this model, we have shown that zDHHC17 interacts with Smad7 and, through palmitoylation, promotes its localization in the cytoplasm, and enhances its stability, thus promoting the inhibitory effect of Smad7 against TGF-β signaling.

In conclusion, we have identified a novel posttranslational modification of the mammalian inhibitory Smad7, which enhances its inhibitory effect on TGF-β signaling.

## Experimental procedures

### Reagents and antibodies

Unless otherwise stated, all chemicals were purchased from Sigma-Aldrich (Sigma-Aldrich AB). Recombinant human TGF-β1 was from PeproTech. Fluorescent 4,6-Diamidino-2-phenylindole dihydrochloride to visualize cell nuclei by microscopy was from Thermo Fisher (Thermo Fisher Scientific). Alkynyl palmitic acid (C16:0) and biotin picolyl azide were bought from Click Chemistry Tools (Scottsdale, AZ 85260). The proteasome inhibitor MG132 was purchased from Millipore (Merck/Millipore) and puromycin was purchased from InvivoGen. Antibodies or antisera against the following proteins were used in immunoblotting (IB), immunoprecipitation (IP), or immunofluorescence (IF) at the indicated dilutions: Flag (KO602, 1:100 for IF) was from TransGenic Inc; HA (Y-11; 1:1000 for IB and 1 μg/mg lysate for IP), GST (B-14; 1:500), c-Myc (9E10; 1:1000 for IB, 1:100 for IF and 1 μg/mg lysate for IP) and ubiquitin (sc-8017, 1:500) were from Santa Cruz Biotechnology; phosho-SMAD2 (1:500) antisera were generated in rabbits in-house (55); antibodies against β-actin (1:10,000), β-tubulin (1:1000), Flag [M2; 1:1000 for IB and lysate (1 μg/mg) for IP] from Sigma-Aldrich AB); GAPDH (14C10; 1:1000), Hsp90 (#4874, 1:1000), LMNB1 (D4Q4Z, 1:1000) from Cell Signaling Technology (Solna, Sweden); horseradish peroxidase-coupled secondary antibodies (1:10,000) from Jackson Immuno Research Laboratories; Alexa Fluor–labeled antibodies (1:1000) for IF were from Invitrogen. Normal mouse immunoglobulin G1 (IgG1) was from R&D Systems (MB002), and normal rabbit IgG from Southern Biotech. Protein A, anti-mouse IgG, streptavidin Dynabeads and anti-HA magnetic beads were from Life Technologies. Thiopropyl Sepharose 6B beads were from GE Healthcare (GE Healthcare).

### Cell culture

Human embryonic kidney (HEK) 293T, human immortalized keratinocyte HaCaT cells, COS-7 green monkey kidney fibroblast, A549 human lung adenocarcinoma, and MDA-MB-231 human breast cancer cell lines were obtained from American Type Culture Collection. HEK293T HaCaT, COS-7, A549, and MDA-MB-231 cells were cultured in Dulbecco’s modified Eagle’s medium (DMEM, Sigma-Aldrich AB), supplemented with 10% (v/v) fetal bovine serum (FBS; Biowest, Almeco A/S), 100 U/ml penicillin and 100 μg/ml streptomycin (Sigma-Aldrich AB). MCF10A cells were obtained from Dr Fred Miller (Barbara Ann Karmanos Cancer Institute) and cultured in DMEM/F12 (Gibco, ThermoFisher Scientific), supplemented with 5% FBS, 20 ng/ml EGF (PeproTech EC Ltd/ThermoFisher Scientific), 100 ng/ml cholera toxin, 0.5 μg/ml hydrocortisone, 10 μg/ml insulin (Sigma-Aldrich AB). All cells were maintained at 37 °C and 5% CO_2_. The cell lines were frequently tested for absence of *mycoplasma* and were authenticated by identity testing.

### Plasmids

Mouse HA-tagged zDHHC family constructs were a kind gift from Dr Masaki Fukata (NIPS) (34). HA-tagged zDHHC17 mutants and GST-ANK17 constructs were a kind gift from Dr Christine Salaun (University of Strathclyde) and described previously (35). pcDNA3 encoding FLAG-Smad7FN (fragment N) and FLAG-Smad7FC (fragment C) were created using PCR-based deletion mutagenesis. The encoded Smad7 fragments correspond to amino acid residues 1 to 261 (Smad7FN), 204 to 426 (Smad7FC), and 204 to 406 (Smad7FCΔ) (56). The expression vectors for HA-tagged TβRI (also called ALK5), 6xMyc-tagged Smad7 proteins (wt, FN, and FC) in the mammalian expression vector pcDNA3 (Invitrogen) were described previously (56, 57). All the Smad7 cysteine mutants described were generated by site-directed mutagenesis. The oligonucleotide primers were designed using QuikChange Primer Design software (Agilent). All plasmids were sequenced by Eurofins Genomics (GATC).

### Lentiviral transduction and transfection

To produce lentiviruses, packaging plasmids (VSV, gag, and Rev) and expression constructs for shRNAs were co-transfected into HEK293T cells. At 48 h post-transfection, supernatants were collected from HEK293T cells and added to target cells supplemented with the same volume of fresh medium. After 48 h of infection, puromycin (1 μg/ml; Sigma-Aldrich AB; P9620) was added to the medium to select stable cells. We used TRCN00000134346 (#2), and TRCN00000134447 (#4) shRNA Clones (Merck Life Science AB) for zDHHC17 knockdown. As a control, an empty pLKO vector was used. For siRNA transfection, 10 nM siGENOME non-targeting siRNA Pool#2 or siGENOME 2 siRNAs targeting human zDHHC17 (MU-014019-01-0002) were transfected into MCF10A cells at 80% confluence with DharmaFECT transfection reagents (Dharmacon/GE Healthcare). The medium was changed at 24 h post-transfection.

### RNA isolation, cDNA synthesis, and quantitative real-time-PCR

Total RNA was isolated by Total RNA Purification Kit (Norgen Biotek Corp). cDNA was prepared by using a High Capacity cDNA Reverse Transcription Kit (Applied Biosystems, ThermoFisher Scientific) using 0.5 μg of total RNA, according to the manufacturer’s instructions. The cDNA samples were diluted 10 times with water. qRT-PCR was performed using 2x qPCR SyGreen Mix (PCR Biosystems) and BioRad CFX96 real-time PCR detection system (Bio-Rad Laboratories AB, Solna, Sweden) according to the manufacturer’s instructions. Relative gene expression was determined using the ΔΔCt method. The expression was normalized to *GAPDH* and quantified relative to the control condition.

### Cycloheximide treatments

At ∼24 h post-transfection, cells were incubated with 20 μg/ml cycloheximide (CHX, Sigma-Aldrich AB). After 0, 4, 8 or 12 h treatments, cells were washed once with PBS, lysed in 2× sodium dodecyl sulfate (SDS) sample buffer containing 25 mM dithiothreitol and heated for 5 min at 95 °C. Samples were processed for immunoblotting as described above. Smad7 protein levels were quantified as a ratio of protein signal (Myc immunoblot) against the corresponding GAPDH.

### Acyl-RAC assay

Protein S-acylation was assessed by the Acyl-RAC assay as described previously (58) with some modifications. In brief, HEK293T cells transfected with Myc- or Flag-tagged Smad vectors were lysed in lysis buffer (1% Triton X-100, 10% glycerol, 20 mM Tris-HCl, pH 7.5, 150 mM NaCl), supplemented with protease inhibitors and aliquots of the cell lysates were directly subjected to SDS–polyacrylamide electrophoresis (SDS-PAGE). Next, 50 mM N-ethylmaleimide was added to the remaining lysates to block free sulfhydryl groups and samples were incubated at room temperature for 2 h. Two hours later, samples were divided into two equal portions: one was treated with 0.75 M hydroxylamine (HA) and the other with 50 mM Tris-HCl, pH 7.4, as a control. After a 2 h incubation at room temperature, activated thiol-Sepharose 6B beads were added and incubated with samples at 4 °C overnight. The Sepharose beads were then washed with 1% SDS in phosphate-buffered saline (PBS) (3 × 2 min), three times in 4 M Urea in PBS, and three times in PBS. Immunoblotting was performed to analyze the presence of Myc- or Flag-tagged proteins.

### Click chemistry

Transfected HEK293T cells were washed in 1 ml of PBS per dish and then incubated with 3 ml/dish of serum-free DMEM containing 1 mg/ml of fatty acid–free bovine serum albumin (BSA) and 100 μM of alkynyl palmitic acid (C16:0) for 4 h at 37 °C and 5% CO_2_. After washing with PBS, cells were lysed on ice using 500 μl/dish of lysis buffer (0.5% SDS, 1% Triton X-100, 10% glycerol, 50 mM Tris, pH 8, 150 mM NaCl, supplemented with protease inhibitor cocktail) and transferred into fresh tubes. For each 100 μl of cell lysate, 80 μl of fresh click reaction mix containing 25 μM of biotin picolyl azide, 2 mM of CuSO4, 0.2 mM of TBTA (Tris[(1-benzyl-1H- 1,2,3-triazole-4yl)-methyl]), and 20 μl of 4 mM ascorbic acid was added. The samples were then vortexed and incubated for 1 h with end-over-end rotation at room temperature. Samples were incubated with Streptavidin DynaBeads (15 μl slurry/sample) overnight. Beads were washed 5 times with lysis buffer, heated at 95 °C for 5 min in SDS-sample buffer, and resolved by SDS-PAGE. Flag-tagged proteins were detected by immunoblotting.

### Immunoprecipitation and immunoblotting

Cells transfected with indicated plasmids were lysed in lysis buffer (1% Triton X-100, 10% glycerol, 20 mM Tris-HCl, pH 7.5, 150 mM NaCl), supplemented with a protease inhibitor mixture Complete (Roche Diagnostics Scandinavia AB) and aliquots of the cell lysates were directly subjected to SDS–PAGE. For immunoprecipitation, lysates were incubated overnight with protein A or anti-mouse IgG Dynabeads that had been preincubated with antibody against target protein or IgG control in PBS supplemented with 0.5% BSA. The beads were washed three times with lysis buffer, and the immunoprecipitated proteins were eluted in SDS-sample buffer and subjected to SDS–PAGE, followed by electrotransfer to nitrocellulose membranes (Amersham Protran, GE Healthcare Life Science) and incubation with antibodies; the chemiluminescent signal was detected using the Clarity Western ECL Substrate (Bio-Rad Laboratories AB).

### Pulldown assay

For GST pulldowns, HEK293T cells expressing the corresponding FLAG-tagged proteins were lysed in lysis buffer (1% Triton X-100, 10% glycerol, 20 mM Tris-HCl, pH 7.5, 150 mM NaCl), supplemented with a protease inhibitor mixture Complete (Roche Diagnostics Scandinavia AB); after clarification by centrifugation (10,000*g* for 10 min at 4 °C), 500 μl of lysates were incubated overnight at 4 °C with GST or GST-fused 17Ank pre-coupled to glutathione-Sepharose resin. After extensive washes with lysis buffer, bound proteins were eluted by boiling in SDS-sample buffer. Following SDS-PAGE on 10% polyacrylamide gels and transfer to nitrocellulose, bound GST proteins were detected by Ponceau S staining and immunoblotting using a FLAG antibody.

### Subcellular fractionation

Cells from a 10-cm dish were collected and lysed in 250 μl of buffer A (1% NP40, 0.25% deoxycholate, 50 mM Tris–HCl, pH 7.4, 150 mM NaCl) for 15 min on ice. After centrifugation at 3000*g* for 5 min, the supernatant was collected and saved as the cytoplasmic fraction. The pellet was washed with PBS twice and resuspended in 150 μl of buffer B (1% SDS, 1% NP40, 0.5% sodium deoxycholate, 50 mM Tris–HCl, pH 7.4, 400 mM NaCl). After 20 min of incubation on ice and centrifugation at 12,000*g* for 15 min, the supernatant was collected and saved as the nuclear fraction.

### Immunofluorescence microscopy

To evaluate the expression and localization of Smad7 (endogenous or Myc-tagged), immunofluorescence staining was performed. In brief, transfected HaCaT cells seeded onto an 8-chamber slide (Falcon) were fixed in 3.7% (w/v) formaldehyde stabilized with 10% (v/v) methanol for 15 min at room temperature. Permeabilization was done with 0.1% v/v Triton X-100 for 10 min followed by blocking with PBS supplemented with 3% FBS for 1 h at room temperature. Subsequently, the cells were incubated with primary antibodies at a dilution of 1:100 in PBS overnight at 4 °C. The next day, the samples were incubated with Alexa Fluor-488 and/or −546-labeled secondary antibody (Invitrogen, Thermo Fisher Scientific) at a dilution of 1:500 in PBS for 1 h at 4 °C. For nuclear staining, DAPI was added to the secondary antibody solution. The samples were examined with a Zeiss Axioplan 2 fluorescence microscope (Carl Zeiss AB). Quantification of nuclear and whole cell Smad7 intensities was performed by Image J software (National Institutes of Health). Briefly, nuclear Smad7 intensity in each cell was quantified based on nuclear segmentation defined by the DAPI channel. Smad7 intensity in each cell (area covered by nucleus and cytoplasm) was quantified as whole-cell Smad7 intensity after cell segmentation based on a visually defined threshold of Smad7. One-way ANOVA followed by Tukey's multiple comparisons or two-tailed student *t* test were performed.

### *In vivo* ubiquitination assay

HEK293T cells transfected with the indicated constructs were treated with 10 μM MG132 for 4 h prior to harvesting. Cells were lysed in radioimmunoprecipitation buffer (1% SDS, 1% NP40, 0.5% sodium deoxycholate, 25 mM Tris–HCl, pH 7.4, 150 mM NaCl, supplemented with protease inhibitors). After heating at 95 °C for 5 min, the lysates were diluted to an SDS concentration of 0.1%. To detect the polyubiquitination of Flag- or 6XMyc-tagged Smad7, cell lysates were incubated overnight with anti-mouse IgG Dynabeads that had been preincubated with the corresponding antibody or IgG control in PBS, supplemented with 0.5% BSA. After five washes, the beads were heated in an SDS-sample buffer and analyzed by immunoblotting.

### Transcriptional reporter assays

To quantify Smad3/4-driven transcriptional CAGA-luc reporter activity (44), MDA-MB231 or COS7 cells were seeded in the wells of a 12-well plate. The next day, cells were transiently transfected with different combinations of reporter constructs and expression plasmids. Total amount of transfected DNAs was the same in each experiment. After 6 h of incubation and serum starvation for 16 h, the cells were stimulated, or not, with TGF-β1 (1 ng/ml) for different time periods. Luciferase activity was measured using the Firefly & Renilla Luciferase Kit (BTIU30003-2, Biotium), the EnSpire plate reader (PerkinElmer Sverige AB), and normalized to β-galactosidase activity. All experiments were performed three times, and representative results are shown.

### Statistical analysis

Data analysis was performed using GraphPad Prism 7.0, and presented as a mean ± standard deviation (SD). Differences were analyzed either with one-way ANOVA followed by Tukey's multiple comparison, or two-sided student unpaired *t* test. In all cases, *p* values equal to or less than 0.05 were considered to indicate a statistically significant result. Essentially all experiments were performed at least three times independently, and similar results were obtained.

## Data availability

The experimental data sets and materials generated and analyzed during the current study are available from the corresponding author upon request.

## Supporting information

This article contains supporting information.

## Conflict of interest

The authors declare that they have no known competing financial interests or personal relationships that could have appeared to influence the work reported in this article.

## References

[1] Heldin C.-H., Miyazono K., ten Dijke P. (1997). TGF-β signalling from cell membrane to nucleus through SMAD proteins. Nature.

[2] Derynck R., Zhang Y.E. (2003). Smad-dependent and Smad-independent pathways in TGF-β family signalling. Nature.

[3] Ten Dijke P., Arthur H.M. (2007). Extracellular control of TGFβ signalling in vascular development and disease. Nat. Rev. Mol. Cell Biol..

[4] Itoh S., ten Dijke P. (2007). Negative regulation of TGF-β receptor/Smad signal transduction. Curr. Opin. Cell Biol..

[5] Nakao A., Afrakhte M., Morn A., Nakayama T., Christian J.L., Heuchel R. (1997). Identification of Smad7, a TGFβ-inducible antagonist of TGF-β signalling. Nature.

[6] Souchelnytskyi S., Nakayama T., Nakao A., Morén A., Heldin C.H., Christian J.L. (1998). Physical and functional interaction of murine and Xenopus Smad7 with bone morphogenetic protein receptors and transforming growth factor-beta receptors. J. Biol. Chem..

[7] Imamura T., Takase M., Nishihara A., Oeda E., Hanai J., Kawabata M. (1997). Smad6 inhibits signalling by the TGF-β superfamily. Nature.

[8] Hanyu A., Ishidou Y., Ebisawa T., Shimanuki T., Imamura T., Miyazono K. (2001). The N domain of Smad7 is essential for specific inhibition of transforming growth factor-β signaling. J. Cell Biol..

[9] Goto K., Kamiya Y., Imamura T., Miyazono K., Miyazawa K. (2007). Selective inhibitory effects of Smad6 on bone morphogenetic protein type I receptors. J. Biol. Chem..

[10] Yan X., Liao H., Cheng M., Shi X., Lin X., Feng X.-H. (2016). Smad7 protein interacts with receptor-regulated Smads (R-Smads) to inhibit transforming growth factor-β (TGF-β)/Smad signaling. J. Biol. Chem..

[11] Ebisawa T., Fukuchi M., Murakami G., Chiba T., Tanaka K., Imamura T. (2001). Smurf1 interacts with transforming growth factor-β type I receptor through Smad7 and induces receptor degradation. J. Biol. Chem..

[12] Kuratomi G., Komuro A., Goto K., Shinozaki M., Miyazawa K., Miyazono K. (2005). NEDD4-2 (neural precursor cell expressed, developmentally down-regulated 4-2) negatively regulates TGF-beta (transforming growth factor-beta) signalling by inducing ubiquitin-mediated degradation of Smad2 and TGF-beta type I receptor. Biochem. J..

[13] Shi W., Sun C., He B., Xiong W., Shi X., Yao D. (2004). GADD34-PP1c recruited by Smad7 dephosphorylates TGFβ type I receptor. J. Cell Biol..

[14] Zhang S., Fei T., Zhang L., Zhang R., Chen F., Ning Y. (2007). Smad7 antagonizes transforming growth factor β signaling in the nucleus by interfering with functional Smad-DNA complex formation. Mol. Cell Biol..

[15] Elkouris M., Kontaki H., Stavropoulos A., Antonoglou A., Nikolaou K.C., Samiotaki M. (2016). SET9-Mediated regulation of TGF-β signaling links protein methylation to pulmonary fibrosis. Cell Rep..

[16] Katsuno Y., Qin J., Oses-Prieto J., Wang H., Jackson-Weaver O., Zhang T. (2018). Arginine methylation of SMAD7 by PRMT1 in TGF-β-induced epithelial-mesenchymal transition and epithelial stem-cell generation. J. Biol. Chem..

[17] Koinuma D., Shinozaki M., Komuro A., Goto K., Saitoh M., Hanyu A. (2003). Arkadia amplifies TGF-β superfamily signalling through degradation of Smad7. EMBO J..

[18] Zhang Y., Chang C., Gehling D.J., Hemmati-Brivanlou A., Derynck R. (2001). Regulation of Smad degradation and activity by Smurf2, an E3 ubiquitin ligase. Proc. Natl. Acad. Sci. U. S. A..

[19] Grönroos E., Hellman U., Heldin C.-H., Ericsson J. (2002). Control of Smad7 stability by competition between acetylation and ubiquitination. Mol. Cell..

[20] Wahl L.C., Watt J.E., Yim H.T.T., De Bourcier D., Tolchard J., Soond S.M. (2019). Smad7 binds differently to individual and tandem WW3 and WW4 domains of WWP2 ubiquitin ligase isoforms. Int. J. Mol. Sci..

[21] Chen B., Sun Y., Niu J., Jarugumilli G.K., Wu X. (2018). Protein lipidation in cell signaling and diseases: function, regulation, and therapeutic opportunities. Cell Chem. Biol..

[22] Chamberlain L.H., Shipston M.J. (2015). The physiology of protein S-acylation. Physiol. Rev..

[23] Salaun C., Greaves J., Chamberlain L.H. (2010). The intracellular dynamic of protein palmitoylation. J. Cell Biol..

[24] Blaskovic S., Adibekian A., Blanc M., Van Der Goot G.F. (2014). Mechanistic effects of protein palmitoylation and the cellular consequences thereof. Chem. Phys. Lipids.

[25] Wegleiter T., Buthey K., Gonzalez-Bohorquez D., Hruzova M., bin Imtiaz M.K., Abegg A. (2019). Palmitoylation of BMPR1a regulates neural stem cell fate. Proc. Natl. Acad. Sci. U. S. A..

[26] Runkle K.B., Kharbanda A., Stypulkowski E., Cao X.J., Wang W., Garcia B.A. (2016). Inhibition of DHHC20-mediated EGFR palmitoylation creates a dependence on EGFR signaling. Mol. Cell..

[27] Sun Y., Li X., Yin C., Zhang J., Liang E., Wu X. (2023). AMPK phosphorylates ZDHHC13 to increase MC1R activity and suppress melanomagenesis. Cancer Res..

[28] Locatelli C., Lemonidis K., Salaun C., Tomkinson N.C.O., Chamberlain L.H. (2020). Identification of key features required for efficient S-acylation and plasma membrane targeting of Sprouty-2. J Cell Sci..

[29] Muszbek L., Haramura G., Cluette-Brown J.E., Van Cott E.M., Laposata M. (1999). The pool of fatty acids covalently bound to platelet proteins by thioester linkages can be altered by exogenously supplied fatty acids. Lipids.

[30] Won S.J., Cheung See Kit M., Martin B.R. (2018). Protein depalmitoylases. Crit. Rev. Biochem. Mol. Biol..

[31] Rana M.S., Lee C.J., Banerjee A. (2018). The molecular mechanism of DHHC protein acyltransferases. Biochem. Soc. Trans..

[32] Li W., Li W., Zou L., Ji S., Li C., Liu K. (2017). Membrane targeting of inhibitory Smads through palmitoylation controls TGF-β/BMP signaling. Proc. Natl. Acad. Sci. U. S. A..

[33] Forrester M.T., Hess D.T., Thompson J.W., Hultman R., Moseley M.A., Stamler J.S. (2011). Site-specific analysis of protein S-acylation by resin-assisted capture. J Lipid Res.

[34] Fukata M., Fukata Y., Adesnik H., Nicoll R.A., Bredt D.S. (2004). Identification of PSD-95 palmitoylating enzymes. Neuron.

[35] Lemonidis K., Gorleku O.A., Sanchez-Perez M.C., Grefen C., Chamberlain L.H. (2014). The Golgi S-acylation machinery comprises zDHHC enzymes with major differences in substrate affinity and S-acylation activity. Mol. Biol. Cell.

[36] Verardi R., Kim J.S., Ghirlando R., Banerjee A. (2017). Structural basis for substrate recognition by the ankyrin repeat domain of human DHHC17 palmitoyltransferase. Structure.

[37] Greaves J., Gorleku O.A., Salaun C., Chamberlain L.H. (2010). Palmitoylation of the SNAP25 protein family: specificity and regulation by DHHC palmitoyl transferases. J. Biol. Chem..

[38] Greaves J., Chamberlain L.H. (2007). Palmitoylation-dependent protein sorting. J. Cell Biol..

[39] Imamura T., Oshima Y., Hikita A. (2013). Regulation of TGF-β family signalling by ubiquitination and deubiquitination. J. Biochem..

[40] Wicks S.J., Grocott T., Haros K., Maillard M., ten Dijke P., Chantry A. (2006). Reversible ubiquitination regulates the Smad/TGF-β signalling pathway. Biochem. Soc. Trans..

[41] Park S.H., Jung E.H., Kim G.Y., Kim B.C., Lim J.H., Woo C.H. (2015). Itch E3 ubiquitin ligase positively regulates TGF-β signaling to EMT *via* Smad7 ubiquitination. Mol. Cells.

[42] Mochizuki T., Miyazaki H., Hara T., Furuya T., Imamura T., Watabe T. (2004). Roles for the MH2 domain of Smad7 in the specific inhibition of transforming growth factor-β superfamily signaling. J. Biol. Chem..

[43] Kamiya Y., Miyazono K., Miyazawa K. (2010). Smad7 inhibits transforming growth factor-β family type I receptors through two distinct modes of interaction. J. Biol. Chem..

[44] Dennler S. (1998). Direct binding of Smad3 and Smad4 to critical TGFbeta -inducible elements in the promoter of human plasminogen activator inhibitor-type 1gene. EMBO J..

[45] Luyckx I., Verstraeten A., Goumans M.-J., Loeys B. (2022). SMAD6-deficiency in human genetic disorders. NPJ Genom. Med..

[46] Slattery M.L., Herrick J., Curtin K., Samowitz W., Wolff R.K., Caan B.J. (2010). Increased risk of colon cancer associated with a genetic polymorphism of SMAD7. Cancer Res..

[47] Monteleone G., Laudisi F., Stolfi C. (2023). Smad7 as a positive regulator of intestinal inflammatory diseases. Curr. Res. Immunol..

[48] Saika S., Ikeda K., Yamanaka O., Miyamoto T., Ohnishi Y., Sato M. (2005). Expression of Smad7 in mouse eyes accelerates healing of corneal tissue after exposure to alkali. Am. J. Pathol..

[49] Casado P., Alcolea M.P., Iorio F., Rodríguez-Prados J.-C., Vanhaesebroeck B., Saez-Rodriguez J. (2013). Phosphoproteomics data classify hematological cancer cell lines according to tumor type and sensitivity to kinase inhibitors. Genome Biol..

[50] Chen S., Zhu B., Yin C., Liu W., Han C., Chen B. (2017). Palmitoylation-dependent activation of MC1R prevents melanomagenesis. Nature.

[51] Hernandez L.M., Montersino A., Niu J., Guo S., Faezov B., Sanders S.S. (2023). Palmitoylation-dependent control of JAK1 kinase signaling governs responses to neuropoietic cytokines and survival in DRG neurons. J. Biol. Chem..

[52] Butler L., Locatelli C., Allagioti D., Lousa I., Lemonidis K., Tomkinson N.C.O. (2023). S-acylation of Sprouty and SPRED proteins by the S-acyltransferase zDHHC17 involves a novel mode of enzyme-substrate interaction. J. Biol. Chem..

[53] Lemonidis K., Sanchez-Perez M.C., Chamberlain L.H. (2015). Identification of a novel sequence motif recognized by the ankyrin repeat domain of zDHHC17/13 S-acyltransferases. J. Biol. Chem..

[54] Itóh S., Landström M., Hermansson A., Itoh F., Heldin C.H., Heldin N.E. (1998). Transforming growth factor beta1 induces nuclear export of inhibitory Smad7. J. Biol. Chem..

[55] Persson U., Izumi H., Souchelnytskyi S., Itoh S., Grimsby S., Engström U. (1998). The L45 loop in type I receptors for TGF-β family members is a critical determinant in specifying Smad isoform activation. FEBS Lett..

[56] Morén A., Imamura T., Miyazono K., Heldin C.H., Moustakas A. (2005). Degradation of the tumor suppressor Smad4 by WW and HECT domain ubiquitin ligases. J. Biol. Chem..

[57] Simonsson M., Heldin C.-H., Ericsson J., Grönroos E. (2005). The balance between acetylation and deacetylation controls Smad7 stability. J. Biol. Chem..

[58] Werno M.W., Chamberlain L.H. (2015). S-Acylation of the insulin-responsive aminopeptidase (IRAP): quantitative analysis and identification of modified cysteines. Sci. Rep..

