# Supplementary material for: Smad7 palmitoylation by the S-acyltransferase zDHHC17 enhances its inhibitory effect on TGF-β/Smad signaling
Source: J Biol Chem. 2024 Jun 12;300(7):107462. doi: 10.1016/j.jbc.2024.107462 (PMC11277750; doi:10.1016/j.jbc.2024.107462)
Supplement: Supporting information [file mmc1.pdf]

Supporting information

Smad7 palmitoylation by the S-acyltransferase zDHHC17 enhances its inhibitory effect on TGF-β/Smad signaling

Oleksandr Voytyuk, Yae Ohata, Aristidis Moustakas, Peter ten Dijke, Carl-Henrik Heldin

Figure S1

Figure S2

Figure S3

Figure S4

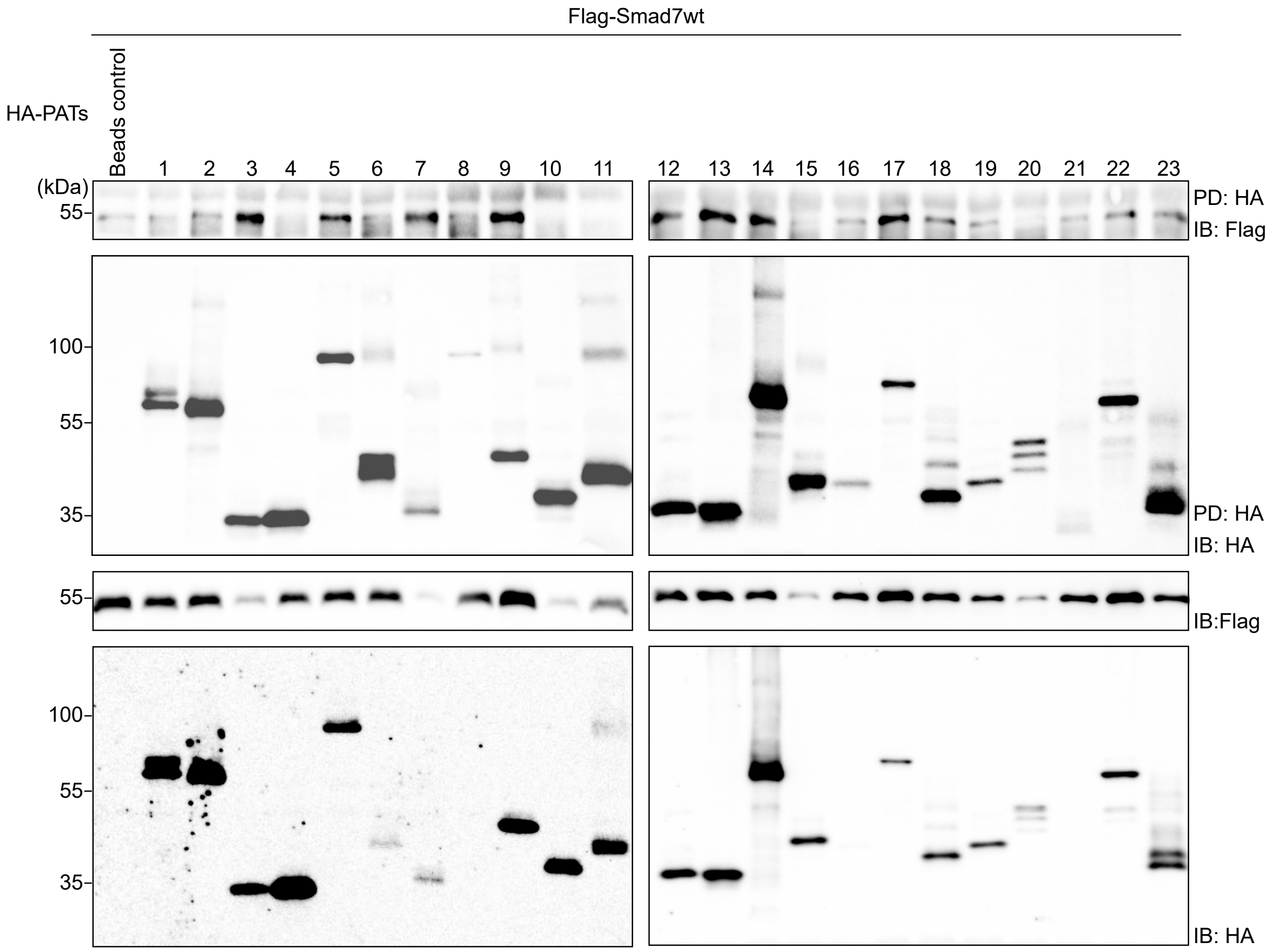

Figure S1. Interaction between members of the zDHHC PAT family and Smad7. Lysates of HEK293T cells transfected with Flag-Smad7 and each of the 23 members of the palmitoyl acyltransferase (PAT) family, were subjected to pull-down with anti-HA magnetic beads (PD), followed by IB using Flag or HA antibodies. The top two panels show the interaction and PD efficiency, and the lower two panels show the expression of each protein, as indicated. The experiment was repeated three times and the result of representative experiment is shown.

A

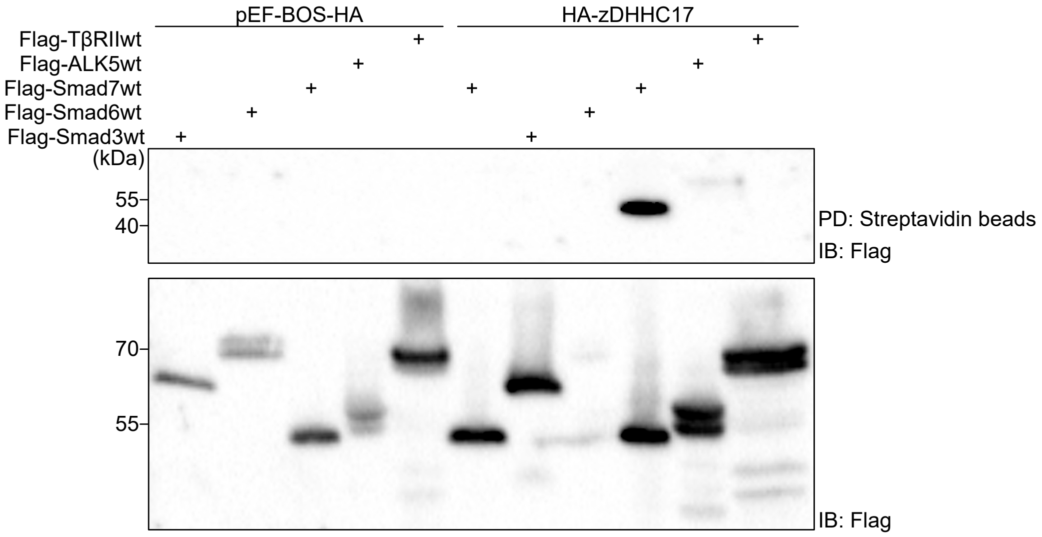

B

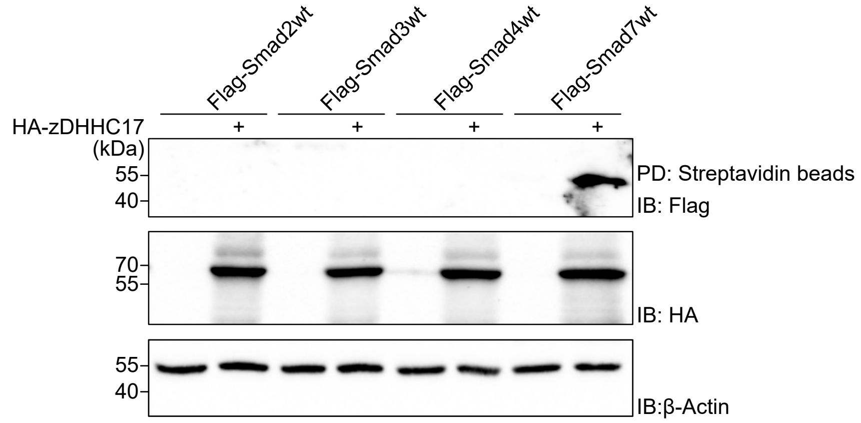

Figure S2. Palmitoylation of TGFβ family members by zDHHC17. *A* and *B,* HEK293T cells were transfected with plasmids encoding the indicated members of the TGF-β family, together with either pEF-BOS-HA (empty vector) or HA-tagged wt zDHHC17. Click reactions were performed and subjected to pull-down with Streptavidin Magnetic beads followed by IB using a Flag antibody. Lower panel (*A*) and two lower panels (*B*) show the expression of each protein. β-Actin was used as loading control. Each experiment was repeated three times and the results of representative experiments are shown.

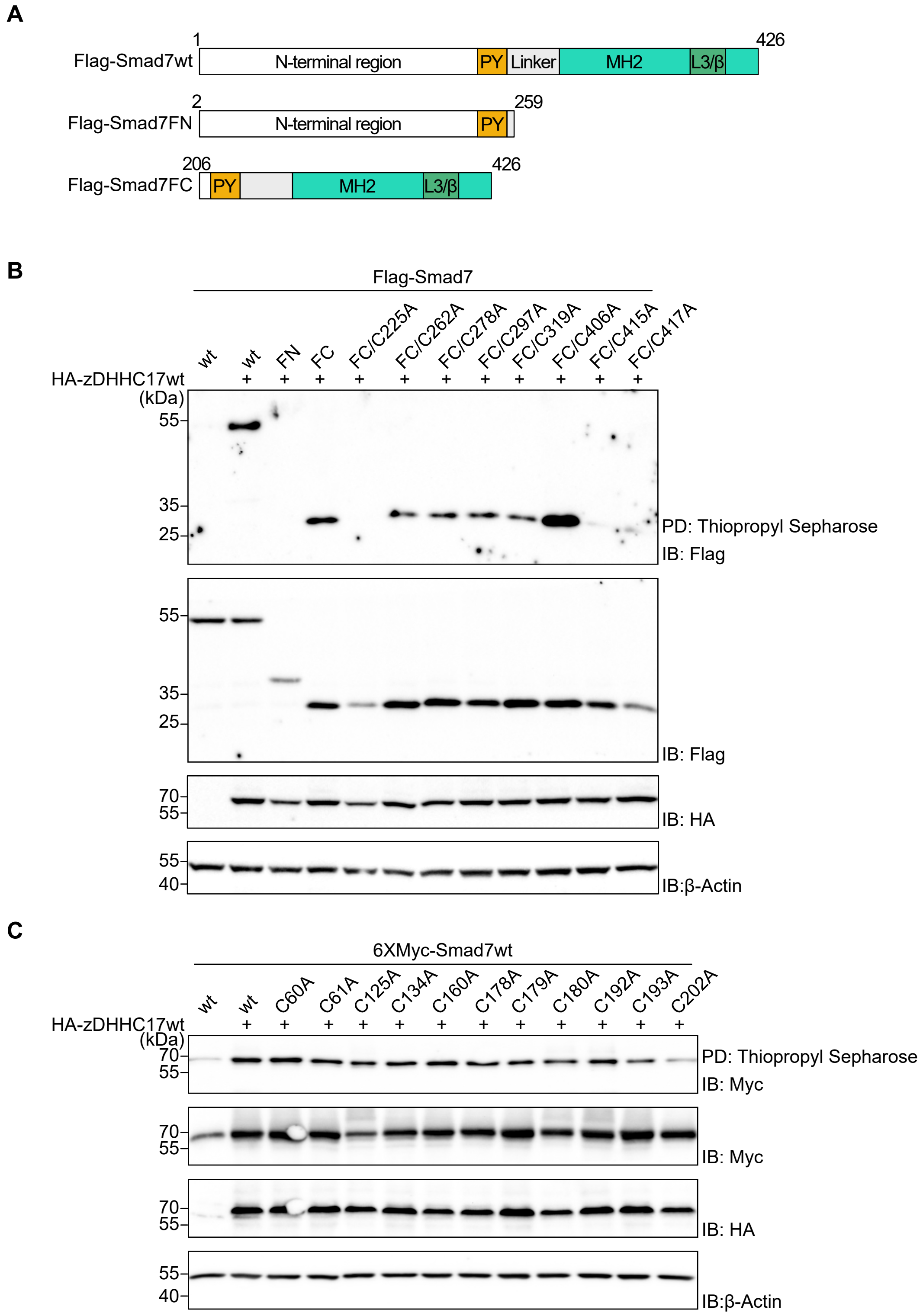

Figure S3. Cysteine residues involved in Smad7 palmitoylation. *A*, Schematic representation of the Smad7 truncation mutant constructs used. *B* and *C*, HEK293T cells were transfected with plasmids encoding truncated parts of Smad7 and Smad7 cysteine mutants, together with HA-tagged wt zDHHC17. Acyl-RAC assays were performed and subjected to pull-down with Thiopropyl Sepharose beads, followed by IB using Flag (*B*) or Myc (*C*) antibodies. Results of Acyl-RAC assays are shown in the upper panels, and the lower three panels show protein expression levels. β-Actin served as loading control. Each experiment was repeated three times and the results of representative experiments are shown.

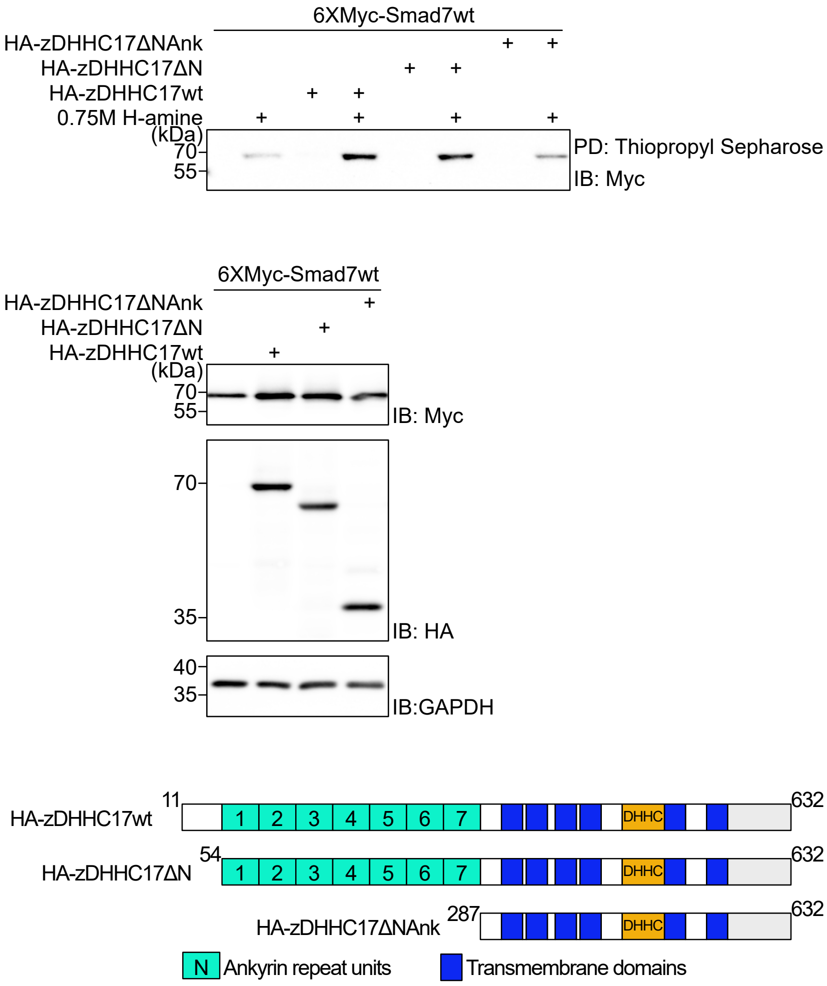

Figure S4. The N-terminal ankyrin repeats of zDHHC17 are important for Smad7 palmitoylation. HEK293T cells were transfected with plasmids encoding the indicated proteins. Cell lysates were analyzed by the acyl-RAC assay, followed by IB using a Myc antibody. Middle panels show protein expression levels and loading control (GAPDH). Schematic figures of the used constructs are shown in the bottom of the figure.The experiment was repeated three times and the result of a representative experiment is shown.
